# Using Transcranial Direct Current Stimulation to Enhance Creative Cognition: Interactions between Task, Polarity, and Stimulation Site

**DOI:** 10.3389/fnhum.2017.00246

**Published:** 2017-05-16

**Authors:** Adam B. Weinberger, Adam E. Green, Evangelia G. Chrysikou

**Affiliations:** ^1^Department of Psychology, Georgetown UniversityWashington, DC, USA; ^2^Department of Psychology, University of KansasLawrence, KS, USA

**Keywords:** creative cognition, transcranial direct current stimulation, idea generation, idea selection, frontotemporal cortex, dorsolateral prefrontal cortex

## Abstract

Creative cognition is frequently described as involving two primary processes, idea generation and idea selection. A growing body of research has used transcranial direct current stimulation (tDCS) to examine the neural mechanisms implicated in each of these processes. This literature has yielded a diverse set of findings that vary depending on the location and type (anodal, cathodal, or both) of electrical stimulation employed, as well as the task’s reliance on idea generation or idea selection. As a result, understanding the interactions between stimulation site, polarity and task demands is required to evaluate the potential of tDCS to enhance creative performance. Here, we review tDCS designs that have elicited reliable and dissociable enhancements for creative cognition. Cathodal stimulation over the left inferior frontotemporal cortex has been associated with improvements on tasks that rely primarily on idea generation, whereas anodal tDCS over left dorsolateral prefrontal cortex (DLPFC) and frontopolar cortex has been shown to augment performance on tasks that impose high demands on creative idea selection. These results highlight the functional selectivity of tDCS for different components of creative thinking and confirm the dissociable contributions of left dorsal and inferior lateral frontotemporal cortex for different creativity tasks. We discuss promising avenues for future research that can advance our understanding of the effectiveness of tDCS as a method to enhance creative cognition.

## Introduction

Creative cognition—cognition manifesting in ideas that are both novel and useful (Barron, 1955; Runco and Jaeger, 2012)—comprises two primary processes: (1) idea generation; and (2) idea selection (Christoff et al., 2001; Smallwood, 2014; Beaty et al., 2016; Chrysikou, in press). Assessments of creativity sometimes examine elements of both of these processes, yet several creativity tasks rely more heavily on one process over the other. Tasks that rely primarily on idea generation involve production of original or unusual responses to presented stimuli. These responses are then assessed on fluency, flexibility and originality (Guilford, 1950). In contrast, tasks that rely primarily on idea selection concern the integration of seemingly remote concepts or pieces of information to discover or identify something novel.

The neuroscientific exploration of creative cognition has focused on brain regions that support creative idea generation and selection using functional neuroimaging and electrophysiological measures. Recent inquiries have also used transcranial direct current stimulation (tDCS) to provide causal evidence for the role of specific brain areas in each of these processes. tDCS is the application of a constant, low-level electrical current to the cortex through surface electrodes positioned on the scalp to modulate the excitability of neurons within a region of interest (Nitsche et al., 2008; Stagg and Nitsche, 2011). tDCS studies may use anodal stimulation (generally intended to increase regional cortical excitability), cathodal stimulation (generally intended to decrease regional cortical excitability), or a combination of the two. Most studies also include a “sham” condition in which electrodes are placed on the scalp but without the application of sustained electrical current. If a cortical target plays a role in creative processing, then modulating activity in that region via tDCS should influence the form of creative thought it supports.

The examination of creative cognition through tDCS has yielded a diverse set of findings that vary depending on the task’s reliance on idea generation or idea selection, as well as the stimulation location and type (anodal, cathodal, or both). Thus, understanding the relationship between task demands and stimulation montages is required to evaluate the potential of tDCS to enhance creative performance. Here, we survey the effects of tDCS on creative cognition, drawing particular distinctions between the generative and selective processes and the corresponding stimulation designs under which enhancements in creative performance can be achieved.

## Creative Idea Generation

Recent theoretical proposals on the neurocognitive mechanisms of creative thinking have suggested that creative idea generation may depend on the availability of unfiltered, low-level perceptual information (e.g., Thompson-Schill et al., 2009; Chrysikou et al., 2014; Chrysikou, 2017). That is, the potential for creative generation is highest when a wider array of possible ideas and solutions to a situation are considered. From this perspective, an effective tDCS design would produce a cognitive mindset that is less inhibited, with a weaker reliance on past routines and representations, allowing for the consideration of information that may have been otherwise prematurely rejected. Researchers have investigated enhancements on forms of creative thought that depend on idea generation by *reducing* cortical excitability of left inferior frontotemporal cortex (a set of regions involved in inhibitory control and semantic knowledge, including the inferior frontal gyrus [IFG], anterior temporal lobe [ATL] and middle temporal gyrus [MTG]) through cathodal tDCS (Chi and Snyder, 2011, 2012; Chrysikou et al., 2013; Mayseless and Shamay-Tsoory, 2015).

**Figure 1:**
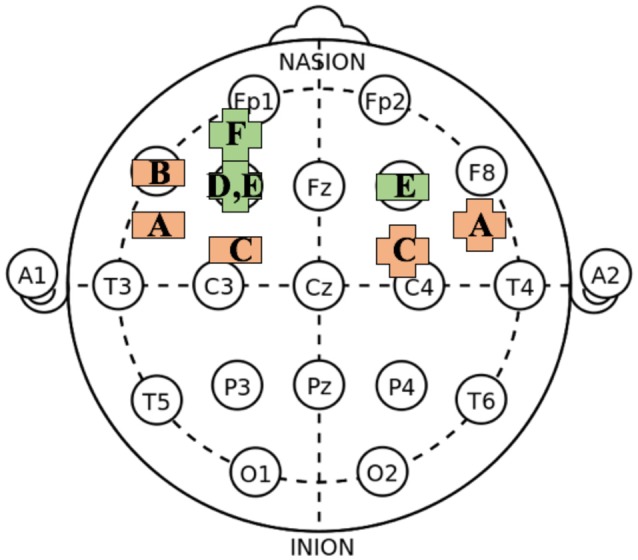
**Approximate transcranial direct current stimulation (tDCS) montage arrangements on International 10–20 system for electroencephalography (EEG) recording associated with increased creative cognition.** (https://commons.wikimedia.org/wiki/File:21_electrodes_of_International_10-20_system_for_EEG.svg); public domain. The figure is a simplification and does not account for differences in montage size/type or duration of stimulation. Plus-symbol = anodal; Horizontal bar = cathodal; Orange = primarily generative tasks; Green = tasks with additional selectivity demands. A = Chi and Snyder (2011, 2012); B = Chrysikou et al. (2013); C = Mayseless and Shamay-Tsoory (2015); D = Cerruti and Schlaug (2009); E = Zmigrod et al. (2015) and Colombo et al. (2015); F = Green et al. (2017).

Chi and Snyder (2011) used bilateral tDCS to target the ATL—a region associated with the storage of mental templates and contexts. They hypothesized that reducing cortical excitability of the left ATL may bring about less reliance on past strategies. Subjects completed challenging insight problems (“matchstick arithmetic”, in which participants are tasked with correcting false statements composed of Roman numerals and symbols formed from matchsticks by moving a stick from one position to another; Ollinger et al., 2008) after solving structurally analogous but conceptually different ones during a pre-testing phase. Such prior exposure has been shown to impair performance on subsequent flexible thinking tasks, likely due to functional fixedness on a routine that was formerly effective but not applicable for the problem participants are currently attempting to solve (e.g., Ollinger et al., 2008). Cathodal stimulation of the left ATL (half way between T7 and FT7 according to the 10/20 electroencephalography (EEG) electrode placement system; Figure 1), with anodal stimulation of the homologous area on the right hemisphere, improved subjects’ performance on the test problems. A follow up study (Chi and Snyder, 2012) produced similar results on the challenging 9-Dot Problem, which requires “thinking outside the box” to connect dots with lines that extend outside the ostensible boundaries of a square array (Maier, 1930); cathodal tDCS over the left ATL with anodal tDCS over the right ATL dramatically improved solution rates for this problem, whereas the opposite stimulation montage did not (Chi and Snyder, 2012). The authors suggested that reducing the excitability of the left ATL might have allowed participants to consider novel approaches as opposed to familiar strategies to solve this problem (see also Goel et al., 2015).

Idea generation has also been successfully augmented through cathodal stimulation of left lateral prefrontal cortex (PFC; Chrysikou et al., 2013). Subjects performed a kind of alternative use task in which they were asked to generate either common (non-creative) or uncommon (creative) uses for a set of everyday objects. Subjects in the uncommon use condition who received cathodal tDCS over left PFC (area F7 on the 10–20 system) generated uses significantly faster and omitted significantly fewer responses than those undergoing cathodal stimulation over the right PFC or sham stimulation. Effects on latencies and omissions were not observed for common uses. These results suggest that a hypofrontal state in which an individual applies less top-down filtering may improve performance on creative generative tasks that rely on unfiltered, bottom-up processing (i.e., generating uncommon uses), but not for tasks that require access to well-rehearsed knowledge (i.e., generating common uses). In line with these findings, Mayseless and Shamay-Tsoory (2015) found that cathodal stimulation of the left IFG with concurrent anodal stimulation of the right IFG significantly improved fluency and flexibility (but not originality) measures on the Alternative Uses Task (AUT) relative to sham stimulation. The reverse montage did not elicit the same effect. A follow up experiment further failed to show significant effects of unilateral cathodal tDCS over left IFG or unilateral anodal tDCS over the right IFG. Thus, in that study, only concurrent cathodal tDCS over the left IFG with anodal tDCS over the right IFG was effective in modulating ideational fluency and flexibility.

Overall, the results of these studies show that reducing the excitability of regions of cortex involved in inhibitory control and the retention of previous experiences and contexts may improve one’s ability to come up with creative ideas or problem solutions. Whether these positive effects on creative cognition also require concurrent excitation of homologous regions in the right hemisphere is likely determined by the nature of the creative task. Although the tasks reported in the current literature primarily involve the generation of creative ideas, they vary with regards to the type of problem solving (i.e., visual, verbal) required or their reliance on the retrieval of semantic information. For example, establishing and breaking a mental set in problems involving visual reasoning was a primary component of the studies by Chi and Snyder (2011, 2012), but was not an element of the experiments by Chrysikou et al. (2013) or Mayseless and Shamay-Tsoory (2015), whose tasks largely relied on verbal semantic memory retrieval. Similarly, the creativity measures (reaction times and omissions) employed by Chrysikou et al. (2013), who inhibited inferior PFC unilaterally, differed from those (fluency, flexibility) used by Mayseless and Shamay-Tsoory (2015), who inhibited left inferior PFC while concurrently exciting right inferior PFC. Lastly, tasks that rely on distancing oneself from current context or an established task mindset may benefit by stimulating temporal cortex (e.g., Chi and Snyder, 2011, 2012), whereas tasks that rely on flexibility in cognitive control (e.g., for memory retrieval) may benefit from stimulating inferior frontal cortex (e.g., Chrysikou et al., 2013; Mayseless and Shamay-Tsoory, 2015). Thus, the effectiveness of particular tDCS montages (e.g., unilateral vs. bilateral; stimulation of temporal vs. inferior frontal cortex) in modulating creative cognition appears to depend on the precise nature of the creativity task used. Despite this variability in the reported effects, overall, these studies support the conclusion that cathodal tDCS over the left inferior frontotemporal cortex can effectively boost performance on creativity tasks that contain a generative component, but have limited—at least not explicit—selection demands.

## Creative Idea Selection

Contrary to creative idea generation, creative idea selection requires task-directed thoughts and integration of semantically distant concepts. When approaching a creative problem, one must be able to effectively direct their thoughts towards a specific goal and evaluate the suitability of potential solutions before choosing the optimal one depending on context and task demands. One appealing neural target for creative thinking that relies on such selective processes is dorsolateral PFC (DLPFC), which has been widely linked to executive function, including promotion of task-relevant thoughts and suppression of inappropriate ones (Bunge et al., 2001; Metzuyanim-Gorlick and Mashal, 2016). The first study to examine the effects of tDCS on creative cognition applied anodal, cathodal and sham stimulation to the left and right DLPFC (F3/F4 on the 10–20 system Figure 1; Cerruti and Schlaug, 2009). The authors assessed creativity via the Remote Associates Test (RAT; Mednick, 1962) in which subjects are presented with three “problem words” and are tasked with identifying the “target word” that links them together (e.g., “Fish, Mine, Rush” → “Gold”). The RAT contains a generative component (subjects must produce a remotely associated word given the three problem words), but its focus on appropriateness places an additional high demand on selectivity (a number of possible solutions connect two of the three problem words, but only one strings together all three; Bowden and Jung-Beeman, 2003; Gonen-Yaacovi et al., 2013). Results indicated that anodal stimulation of left, but not right, DLPFC selectively improved RAT scores without affecting solution latencies. Although additional research is required to better understand the lateralized effects, the results are consistent with the well-established role of DLPFC in guiding task-appropriate thoughts—a cognitive process that is relevant for idea selection. Increasing regional excitability of left DLPFC produced gains on a task that required not only generation, but also selection of creative ideas. The non-significant outcome of cathodal tDCS to the same area further supports this conclusion; boosts following a *reduction* of excitability of left DLPFC would have been antithetical to theories that implicate this region in creative idea selection. Critically, enhancements did not extend to a separate verbal fluency task, a measure of creativity that is primarily generative. Increased DLPFC activity may not be enough to induce changes in performance on tasks that rely more heavily on idea generation, suggesting that other regions (e.g., inferior PFC) have a more critical role in highly generative forms of creative thought.

### The Thinking Cap Effect

Research has revealed that humans are able to deliberately think more creatively when prompted by explicit instructions to do so (Harrington, 1975; Chen et al., 2005; O’Hara and Sternberg, 2011; Green et al., 2012a; Nusbaum et al., 2014; Weinberger et al., 2016). These findings suggest that—beyond being a stable *trait* that differs among individuals—creativity is also a *state* that can vary acutely over time. Functional neuroimaging has shown that enhancement of this creative state is associated with increased activation and altered functional connectivity of left frontopolar cortex during creative verbal relational thinking (Green et al., 2010, 2012b, 2015; Prabhakaran et al., 2013). Furthermore, the formation of more creative analogies has been associated with greater activation of the same region (Green et al., 2012a; Prabhakaran et al., 2013). For tasks of creative relational thinking, creativity of responses is typically assessed by “semantic distance”—a measurement of similarity/difference between the English-language context usages of terms that form analogies (more creative analogies cover a greater semantic distance; Green, 2016). Frontopolar cortex is also a good candidate to support creative idea selection because of its well-established role in more broad cognitive processes; following the rostro-caudal hierarchy of prefrontal cognitive architecture, frontopolar cortex is likely engaged in combining abstract pieces of information (Badre and D’Esposito, 2009). Additionally, neurons in frontopolar cortex are highly arborized, suggesting a key role in integrating abstract representations (Ramnani and Owen, 2004; Knowlton et al., 2012). As such, potentiating this area with anodal tDCS should produce gains on one’s ability to combine and evaluate semantically distant information during analogical reasoning, thus improving performance on creativity tasks that require idea selection. To explore this prediction, Green et al. (2017) recently used anodal tDCS to target the region of peak activation of left frontopolar cortex observed in the foregoing neuroimaging studies (AF3 on the 10–20 system). Following stimulation, participants completed: (1) a task in which they were presented with word pairs (i.e., Helmet: Head) and were explicitly cued to think creatively as they selected additional word pairs from a large matrix to form valid and creative analogies (Atmosphere: Earth); and (2) a verb generation task in which they saw noun prompts onscreen and generated verbs that were related to the nouns (i.e., see: “arrow” → say: “shoot”), with a cue to think creatively given on half of the trials (Green et al., 2017). Anodal stimulation of left frontopolar cortex relative to sham lead to significantly improved creative performance on the matrix search task (as measured by semantic distance between word pairs), and a tDCS × Creativity Cue interaction yielded maximal creative performance on the verb generation task. These results are consistent with past literature on other aspects of cognition for which the combined effects of stimulation and behavioral interventions (i.e., cuing, priming) that engage the same structure targeted by tDCS have yielded larger effect sizes compared to tDCS alone (e.g., Jacobson et al., 2012).

In the absence of a creativity cue, the analogy matrix and the verb generation tasks depend on both creative processes, although the former task likely places a greater demand on selection (participants, quite literally, select appropriate creative word pairs), whereas the latter task more strongly taxes the generative resources. However, instructing participants to think creatively introduces additional selectivity demands—particularly for the verb generation task. When participants were asked to think creatively, they needed to inhibit more common, prepotent responses and select more semantically distant options. With this greater focus on idea selection, potentiating left frontopolar cortex produced greater enhancements compared to the uncued—and less selective—conditions. Our study showed that, without a cue to think creatively, anodal tDCS to left frontopolar cortex was not associated with enhanced performance on the otherwise non-selective verb generation task (see also Brunye et al., 2015). Thus, only after emphasizing selectivity explicitly did potentiating left frontopolar cortex boost performance, suggesting support for the region’s contributions to creative idea selection.

Similarly, anodal stimulation to left DLPFC, paired with cathodal stimulation to right DLPFC, can improve remote association performance compared to the reverse stimulation montage or no stimulation; yet the same montage did enhance performance on the AUT, a largely generative measure as discussed above (Zmigrod et al., 2015). However, pairing the same stimulation design with explicit instructions to visualize using an object in an unusual, relative to its typical, way significantly elevated AUT total creativity scores (Colombo et al., 2015). In line with the findings of Green et al. (2017), these results demonstrate that when participants deliberately search for more creative or unusual responses the need for selectivity is amplified. Critically, anodal tDCS over regions implicated in directing one’s thoughts towards a specific goal led to enhanced creativity after increased selection demands.

Taken together, this emerging literature suggests that anodal tDCS over cortical areas involved in promoting relevant thoughts and integrating discrete pieces of information (DLPFC and frontopolar cortex, respectively) can augment creative idea selection. These results support the effectiveness of this particular study design in which stimulation type (anodal) and location (left DLPFC, left frontopolar cortex) interact with task context (increased demands on selectivity) to produce significant behavioral gains for creativity performance.

## Conclusion

Creative cognition likely relies on two primary operations, idea generation and idea selection. Although most measures of creative thought involve—to an extent—both of these processes, the growing literature on tDCS interventions to promote creative thinking suggests that creative idea generation and idea selection involve inherently different mechanisms with distinct neural bases. This review article outlined tDCS designs that have elicited dissociable creative enhancements on each of these processes. Cathodal stimulation over the left inferior frontotemporal cortex, a region implicated in inhibitory control and the maintenance of mental templates, has been associated with improvements on tasks that rely primarily on idea generation, without significantly changing performance on tasks that rely primarily on idea selection. In contrast, anodal tDCS over left DLPFC and frontopolar cortex—brain regions that likely contribute to goal-directed thought and informational integration—can augment performance on tasks that impose high demands on creative idea selection, without significant consequences for tasks that rely primarily on creative idea generation.

tDCS effects on creative cognition as a function of the interactions between task, polarity and stimulation site highlight a critical aspect of the *in vivo* neurobiological mechanisms of tDCS: the effects of tDCS may be functionally specific, because the stimulation may affect mechanisms that are already undergoing neural plasticity (Reato et al., 2010; Rahman et al., 2015). As the contributions of left dorsal and inferior lateral frontotemporal cortex vary by the nature of the creative task (i.e., generative vs. selective), so does the effectiveness of excitatory or inhibitory stimulation over these regions. Based on the current literature, the particular montages detailed above are anticipated to elicit positive effects on creative performance depending on the generation or selection emphasis of the creative task. Nevertheless, several questions still remain. What are the neurochemical mechanisms underlying tDCS effects for creative thinking? How do individual differences due to expertise or individual genetic variability influence the effectiveness of electrical stimulation? Under which circumstances does bilateral stimulation benefit performance in creativity tasks? Extensive examination of these and other questions in future research will advance our understanding of the effectiveness of tDCS as an intervention that can reliably augment creative cognition.

## Author Contributions

ABW, AEG and EGC co-designed the project; wrote the manuscript. AEG and EGC provided conceptual guidance on the manuscript.

## Funding

EGC is supported by an Advancing the Science of Imagination grant #50696 from the Imagination Institute. ABW and AEG are supported by grants from the National Science Foundation (DRL-1420481) and The John Templeton Foundation (ID 51971).

## Conflict of Interest Statement

The authors declare that the research was conducted in the absence of any commercial or financial relationships that could be construed as a potential conflict of interest.
